# Immune modulations of the tumor microenvironment in response to phototherapy

**DOI:** 10.1142/s1793545823300070

**Published:** 2023-04-27

**Authors:** Trisha I. Valerio, Coline L. Furrer, Negar Sadeghipour, Sophia-Joy X. Patrock, Sayre A. Tillery, Ashley R. Hoover, Kaili Liu, Wei R. Chen

**Affiliations:** * Stephenson School of Biomedical Engineering University of Oklahoma, Norman, Oklahoma 73019, USA; † School of Electrical and Computer Engineering University of Oklahoma, Norman, Oklahoma 73019, USA

**Keywords:** Phototherapy, immunotherapy, tumor microenvironment, tumor-infiltrating lymphocytes, phototherapy-induced tumor immunogenic cell death, translational combination therapies

## Abstract

The tumor microenvironment (TME) promotes pro-tumor and anti-inflammatory metabolisms and suppresses the host immune system. It prevents immune cells from fighting against cancer effectively, resulting in limited efficacy of many current cancer treatment modalities. Different therapies aim to overcome the immunosuppressive TME by combining various approaches to synergize their effects for enhanced anti-tumor activity and augmented stimulation of the immune system. Immunotherapy has become a major therapeutic strategy because it unleashes the power of the immune system by activating, enhancing, and directing immune responses to prevent, control, and eliminate cancer. Phototherapy uses light irradiation to induce tumor cell death through photothermal, photochemical, and photo-immunological interactions. Phototherapy induces tumor immunogenic cell death, which is a precursor and enhancer for anti-tumor immunity. However, phototherapy alone has limited effects on long-term and systemic anti-tumor immune responses. Phototherapy can be combined with immunotherapy to improve the tumoricidal effect by killing target tumor cells, enhancing immune cell infiltration in tumors, and rewiring pathways in the TME from anti-inflammatory to pro-inflammatory. Phototherapy-enhanced immunotherapy triggers effective cooperation between innate and adaptive immunities, specifically targeting the tumor cells, whether they are localized or distant.

Herein, the successes and limitations of phototherapy combined with other cancer treatment modalities will be discussed. Specifically, we will review the synergistic effects of phototherapy combined with different cancer therapies on tumor elimination and remodeling of the immunosuppressive TME. Overall, phototherapy, in combination with other therapeutic modalities, can establish anti-tumor pro-inflammatory phenotypes in activated tumor-infiltrating T cells and B cells and activate systemic anti-tumor immune responses.

## 1. Introduction

Cancer is the second leading cause of death^1^ and an important barrier to increase the life expectancy worldwide.^2^ Conventional cancer treatments include surgery, chemotherapy, and radiation. The treatments are based on the stage of cancer aiming to cure or stop the disease from spreading further. However, fighting cancer is not an easy task, particularly because of tumor metastasis and tumor immune escape. Approximately 90% of cancer deaths are caused by metastatic cancer.^3,4^ Many types of cancers can resist conventional therapies because tumors escape from the host’s immune system.^5,6^ Therefore, different combinations of drugs and therapies are usually the most promising way to destroy tumor cells.^7,8^ New strategies based on cutting-edge therapies, such as immunotherapy, gene therapy, phototherapy, and nanomedicine are being developed and tested. These innovative therapies progressively arrive in clinical practice and on the market, presenting numerous promises and challenges. One of the limitations of these new strategies is low efficacy due to the patients’ weakened immune system, anemia, nausea, fatigue, etc.^9^ Hence, studies to shed light on the effects of advanced treatment modalities on tumor immune escape and the immunosuppressive tumor microenvironment (TME) are urgently needed.

The TME is a rich and complex environment, including immune cells, stromal cells, extracellular matrix, and surrounding blood vessels, which modulates tumor growth and metastasis. The wide variety of components in the TME plays a crucial role in therapeutic efficacy since this environment is pro-tumor and mainly anti-inflammatory. The abundance and infiltration of immune cells in the TME depends on the tumor type and are known to be significant prognostic factors in cancer treatment.^10^ To overcome the immune suppression of the TME, phototherapy, especially photothermal therapy (PTT), combined with nanomedicine has become a viable option. PTT disrupts tumor homeostasis, which leads to the release of tumor antigens and danger-associated molecular patterns (DAMPs). Nanoparticles (NPs) not only allow for local application of the phototherapeutic effect but can also be functionalized by immune stimulants, TME modulators, and/or chemotherapeutic agents to significantly enhance anti-tumor immune stimulation and tumor killing. Synergizing phototherapy with immunotherapy is a viable option to overcome the current limitations of cancer therapeutics. Immunotherapy has become a major cancer treatment modality because it unleashes the power of the immune system by activating, enhancing, and directing immune responses to prevent, control, and eliminate cancer. Combining phototherapies with nanomedicine and immunotherapy can greatly enhance their therapeutic effects by rewiring the immunosuppressive activities in the TME.

In this review, we will present major components of immunity in the TME and how they limit tumoricidal activity. We will also discuss the successes and limitations of current treatments targeting the TME. Furthermore, phototherapy-induced immune modulations will be examined. Specifically, we will herein describe the T and B lymphocyte modulations in various cancer models and present the correlations between immune modulations and cancer patient survival.

## 2. Tumor Microenvironment

### 2.1. TME at a glance

The TME is a corrupted ecosystem independent of the biology of the body.^11^ Composition and architecture of the TME are unique and different from any healthy tissue. The TME of a solid tumor is characterized by a low pH, hypoxia, acidosis, and high interstitial pressure.^12^ Cellular and acellular components of the TME can reprogram tumor initiation, growth, invasion, and metastasis. A solid tumor is composed of malignant parenchyma and stromal cells that support the tumor structure and growth.^13^ In the environment of a solid tumor, the vasculature is different from the vasculature in healthy tissues and is highly heterogeneous.^14,15^ The structure of the vasculature surrounding a tumor is specialized for providing increased supplies of oxygen and nutrients to the tumor. Diverse noncellular components such as extracellular matrix, cytokines, and multiple signaling molecules involved in numerous communication pathways between the cells are present in TME.^16^ Different types of cells have been identified in the TME, such as cancer-associated fibroblasts (CAFs),^17^ malignant cells, necrotic and hypoxic cells,^18^ stem cells,^19^ vascular endothelial cells,^20^ adipocytes,^21^ and pericytes.^22^ Immune cells in the TME are major players in tumor response to treatments.^23^

**Fig. 1.**
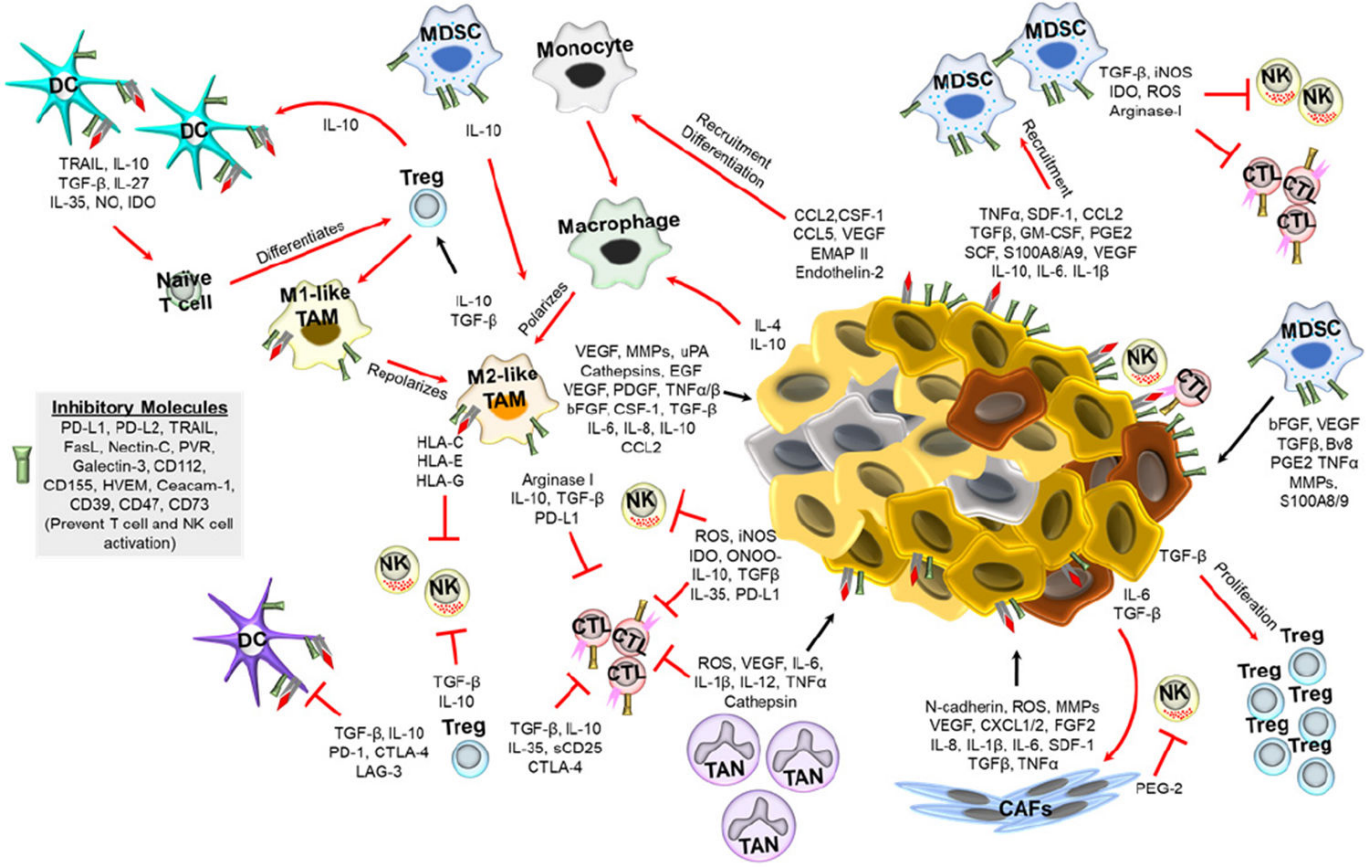
The TME and interplays between tumor and immune cells.^26^ In the TME, tumor cells and immune cells are in constant communication. The TME is acidic, nutrient deficient, and hypoxic, which promotes an immunosuppressive environment. Additionally, the tumor cells and cancer associated fibroblasts (CAFs) secrete factors that promote tumor growth, inhibit immune cell activation, and recruit immunosuppressive immune cells. The immunosuppressive cells, such as M2-polarized macrophages (M2-like TAMs), myeloid derived suppressor cells (MDSCs), T regulatory (Treg) cells, and tumor associated neutrophils (TANs), not only suppress anti-tumor immunity directly (via expression of inhibitory receptors) or indirectly (cytokines, chemokines, etc.), but also promote tumor growth and metastasis through various mechanisms such as inhibiting DC, NK cell, and cytotoxic T lymphocyte (CTL) functions.

The TME encompasses multiple types of immune cells: Tumor-associated neutrophils (TANs), tumor-associated macrophages (TAMs), dendritic cells (DCs), myeloid-derived suppressor cells (MDSCs), mast cells, granulocytes, T lymphocytes, T-regulatory cells (Tregs), B cells, and natural killer (NK) cells.^24^ Immune cells in the TME are distributed into three types: Immune infiltrated, immune excluded, and immune silent.^25^ Figure 1 shows the interplay of tumor and immune cells in the TME as well as the main physiological conditions in this environment.

The composition of a tumor and TME determines how cancer patients respond to therapies such as radiation therapy and/or chemotherapy^27,28^ as well as immunotherapy.^29^ The TME can reprogram tumor initiation, growth, invasion, and metastasis by transforming infiltrating cells to help maintain tumor homeostasis and promote the survival of cancer cells.^23^ In response to signals from the tumor, various immune cells are recruited to the tumor site, often having their anti-tumor functions inhibited and being stimulated to promote tumor growth while anti-tumor effector cells die by apoptosis, leading to the tumor escaping from the host’s immune system.^30^ Various components actively interact with one another and interplay in the complexity of the TME (Fig. 1). Overall, the TME participates in immune evasion, tumor escape, hypoxia, acidosis, metabolic exchange, local invasion of distant locations (metastasis), angiogenesis, and tumor growth.

The suppressive immune microenvironment that helps cancer to avoid immune destruction is closely related to the cancer prognosis.^31-34^ A better understanding of cellular and molecular pathways involved in the immune escape mechanisms in the TME would allow cancer researchers to develop effective strategies to block tumor escape.^35^ Thus, novel therapies are designed to change the pro-TME to one favoring acute responses and potent anti-tumor activity.^36-38^ Since composition and morphology of the TME strongly affect how tumors respond to therapies, a variety of parameters should be included in models simulating tumor growth and responses to cancer treatment modalities. Those data are used to determine the most effective treatment regimens depending on tumor properties in order to improve, personalize, and customize treatment plans for cancer patients. Thus, accurate simulations of the TME are important in exploring and optimizing targeted cancer treatment techniques.^39^

### 2.2. Cancer immunoediting

Cancer immunity plays a key role in tumor progression. Cancer immunoediting describes the mechanism by which the immune system responds to cancer and vice versa.^40^ This mechanism can be divided into four phases. The first phase is the *initiation* phase, when cancer starts to develop. The second phase is *elimination*, which corresponds to immunosurveillance, where the immune system recognizes and attacks tumor cells. Several immune cells are involved in tumor elimination, such as T cells, B cells, and NK cells. Later, we will describe the mechanisms of immunosurveillance. After elimination comes the *equilibrium* phase, when some tumor cells can evade immune recognition, survive, and grow. At this point, immune cells can no longer kill cancer cells and the most aggressive tumors overcome the host anti-tumor immunity. Phase four is the *escape* phase when the tumor cells that escaped the immune system proliferate in an uncontrolled manner and invade surrounding tissues and distant organs.

### 2.3. Tumor-infiltrating monocytes

Immune cells such as DCs and TAMs are crucial to control TME development since they are involved in immunosurveillance, and more precisely, in tumor detection and tumor-induced immune responses. Understanding the immunostimulatory and immunosuppressive effects of tumor-infiltrating monocytes is crucial to identify the connection between innate and adaptive immune responses.

DCs are major surveillance cells in the TME and are divided into heterogeneous populations but their main function is to uptake and present antigens, migrate to the lymph nodes, and present antigens to prime naive T cells into effector T cells.^41^ DCs in the TME are involved in the workings behind radiation therapy and chemotherapy. However, vaccination with DCs has not been consistently achieved yet due to the variety of tumor antigens and the immunosuppressive TME.^42^ In the environment of a tumor, DCs may be polarized into immunosuppressive DCs which diminish their antigen-presenting function and allow for cancer escape. The largest obstacle to an effective immune response of DCs is the presence of Tregs, as they mutually inhibit each other.^43^ To avoid DC’s phenotype to be rewired from anti- to pro-tumor in the TME, more studies on DC maturation, migration, and specialization processes are needed to determine how DCs can enhance anti-tumor immune activities.

Macrophages start their life cycle as monocytes from the bone marrow. Once monocytes are activated by a pathogen, they can mature into macrophages. Mature macrophages can take on many roles from wound repair, tissue maintenance and development, degradation of cellular debris through phagocytosis, to immune surveillance and immediate defense against outside invaders.^25,44^ Macrophages are primarily recruited to tumors to acquire a pro-tumorigenic phenotype (M2-like) due to the TME’s hypoxic conditions and secretion of cytokines such as IL-4.^45^ M2-polarized macrophages promote tumor growth, angiogenesis, metastasis, and immunosuppression.^41^ If not modulated by the TME, macrophages will go into the M1-like phenotype, where they support the anti-tumor activity of the immune system as well as the propagation of pro-inflammatory immune cells and tissue-remodeling molecules.^46^

### 2.4. Tumor-infiltrating lymphocytes

Tumor-infiltrating lymphocytes (TILs) are major immune cells that infiltrate the TME in response to various treatments.^47,48^ TILs are divided into three main immune cell populations: T cells, B cells, and NK cells. Subsets of T lymphocytes include CTLs, helper T cells, and Tregs. Subsets of B lymphocytes include antigen-presenting B cells, antibody-producing B cells, and regulatory B cells.

CTLs or CD8^+^ T cells are effector cells specialized in immunosurveillance and tumor cell killing.^49^ The CTLs’ adaptive immune response involves direct tumor cell killing via secretion of cytokines, perforins, and granzymes. When some tumor cells start to undergo CTL-mediated apoptosis, tumor-specific antigens are generated. Those antigens are internalized and presented at the surface of antigen-presenting cells (APCs), such as macrophages and DCs. When resting APCs encounter tumor-specific antigens, they mature and migrate to the lymph nodes, where they cross-present the tumor-specific antigens to naïve T cells in the germinal center.^50^ CD8^+^ T cells are therefore primed and activated against the specific tumor where the antigens come from. Activated T cells proliferate and migrate to the tumor site, where their CTL activity causes tumor cell death, further generating antigens and maintaining the tumor-specific T cell induction and anti-tumor function.^51^ When the CTLs successfully interact with the MHC-I molecule of the target tumor cell, molecules like perforins and granzymes kill the tumor cell through the formation of pores in the membrane of the target cell. Effector T cells also produce cytokines such as interferon-gamma (IFN-*γ*) and tumor-necrosis factor alpha (TNF-*α*) leading to tumor cell apoptosis. When the tumor cell is dying from damages caused by the CD8^+^ T cell, the receptors of the two cells separate from each other and the T cell is released.^52^ Hence, DCs play a crucial role in generating anti-tumor CTL immunity.

CD4^+^ T helper lymphocytes are adaptive immune cells that have a central role in anti-tumor immune response and can kill tumor cells through two pathways^53^: (1) Direct tumor killing in the case that CD4^+^ T helper cells are tumor antigen-specific and bind to MHC Il-expressing tumor cells. In addition, helper T cells can directly kill cancer cells by secreting tumor-killing molecules; (2) Indirect tumor killing in the case that CD4^+^ T helper cells activate tumor-infiltrating macrophages, which in turn eliminate tumor cells. CD4^+^ T lymphocytes are multifunctional and can differentiate into several subtypes in response to specific signals. Upon presentation of antigens, naive CD4^+^ T cells can differentiate into Th1 (T helper 1), Th2 (T helper 2), Th17 (T helper 17), Tfh (T follicular helper), and Treg cells. These subtypes of CD4^+^ T cells have distinct immune functions and cytokine profiles.^54^ Th1 cells, for instance, inhibit angiogenesis and promote the priming and recruitment of CD8^+^ T cells and NK cells to regulate tumor cell killing. Th2 cells participate in the recruitment of eosinophils or tumor-promoting activities. A combination of transforming growth factor beta (TGF-*β*) and interleukin-6 (IL-6) induces Th17 cell activation, which results in the promotion of angiogenesis and tumor progression, but the effects of Th17 cells remain unclear. Finally, Tfh cells have been shown to enhance anti-tumor associated antigen responses, produce IL-21 to stimulate and activate B cells, and play a key role in immune cell recruitment, which consequently, results in positive prognosis.^55-58^

Tregs are another subtype of CD4^+^ T lymphocytes, and their function is to suppress excessive immune responses in order to maintain homeostasis. Tregs infiltrate the TME in response to chemokine gradients such as CCR10-CCL28, CCR8-CCL1, and CCR4-CCL17/22.^59,60^ The recruitment, activation, and proliferation of Tregs contribute to the establishment of an immunosuppressive TME. High Treg infiltration correlates with poor patient survival in several types of cancer. For example, Plitas *et al.* found that patients with high CCR8^+^ FoxP3^+^ Treg infiltration had significantly lower survival rate than those with lower Treg infiltration in the tumor.^61^

Although targeting T cells for cancer immunotherapy has been extensively described in previous work, only several studies suggest a positive correlation between the presence of B cells in the TME and improved prognosis in various cancer types.^62-64^ Regulatory B (Breg) cells indirectly suppress immune responses by secreting the following cytokines: IL-10, IL-35, and TGF-*β*. Bregs can also directly suppress anti-tumor immunity by inhibiting effector cells such as CTLs. Other subtypes of B lymphocytes include antigen-presenting B cells and antibody-producing B cells (plasma cells). As APCs, B cells can efficiently present tumor antigens to T cells and promote anti-tumor immune responses. Antibodies produced by B cells specifically bind to tumor cells, which enhances tumor recognition and elimination by the immune system. B cells can also secrete immunostimulatory cytokines such as IFN-*γ* and release granzymes, resulting in cytotoxic anti-tumor immune responses, but this phenomenon is still not fully understood.^47,65^

Along with T cells and B cells, NK cells are effector lymphocytes that make up the trifecta of immune cells in the adaptive immune response to cancer. NK cells are cytotoxic cells that can identify and destroy tumor cells.^66^ NK cells can fight tumors directly and produce pro-inflammatory cytokines, leading to tumor cell death. More attention has been given to NK cell functions in immunotherapy due to their ability to effectively kill tumor cells.^67^

**Fig. 2.**
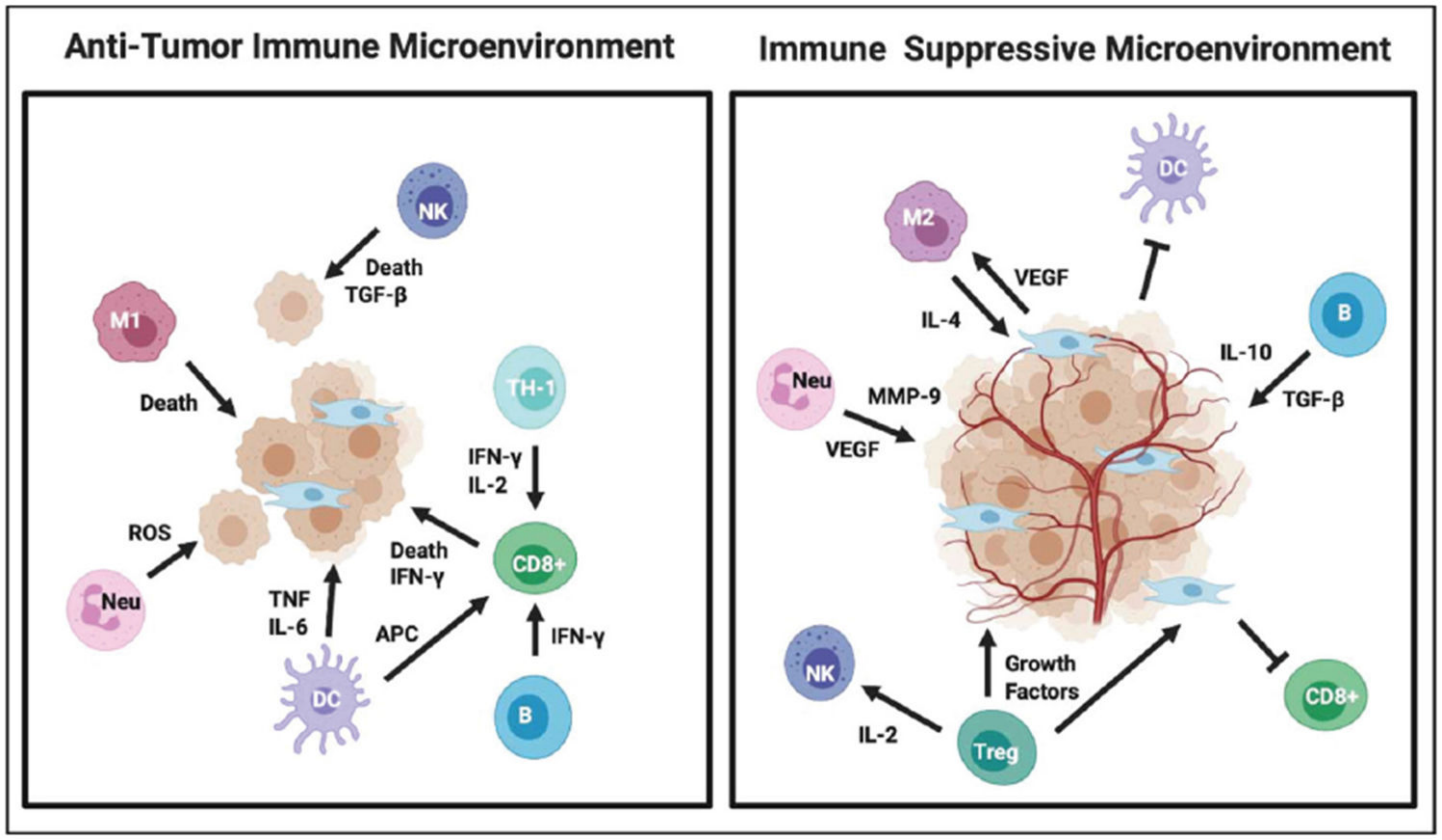
Immune cells in the TME can either suppress tumor formation (anti-TME) or promote tumorigenesis (pro-TME). Depending on context and tumor type, immune cells can both be pro- or anti-tumorigenic.^25^

Overall, the TME is a very complex ecosystem since tumor-infiltrating immune cells can have a pro- or anti-tumorigenic phenotype, as summarized in Fig. 2. Cancer and immunology are complexly interconnected; they adapt and evolve together with tumor progression. The TME is complex and unique to each individual tumor, even in the same patient. Therefore, multimodal, personalized therapeutic approaches are required to unleash the power of the immune system to kill tumor cells.

## 3. Phototherapy-Modulated TME for Cancer Treatment

Fighting cancer is challenging because patients’ immune system often fails to recognize and fight tumor cells, especially because of the immunosuppressive TME. Many therapeutics have been developed to fight against cancer. Conventional therapeutics include surgery which is commonly used to remove solid tumors, alongside anti-tumor drugs and radiation that have been the standard of treatment in many cases. Recently, immunotherapy has become an important cancer treatment modality and is now the first choice for many patients, especially when used in combination with other therapeutics.^68-70^ Laser irradiation has emerged as a useful cancer treatment option since light can harness photothermal and photodynamic effects to treat cancer.^71,72^

### 3.1. Phototherapy combined with nanomedicine modulates the TME

#### 3.1.1. Photodynamic therapy

**Fig. 3.**
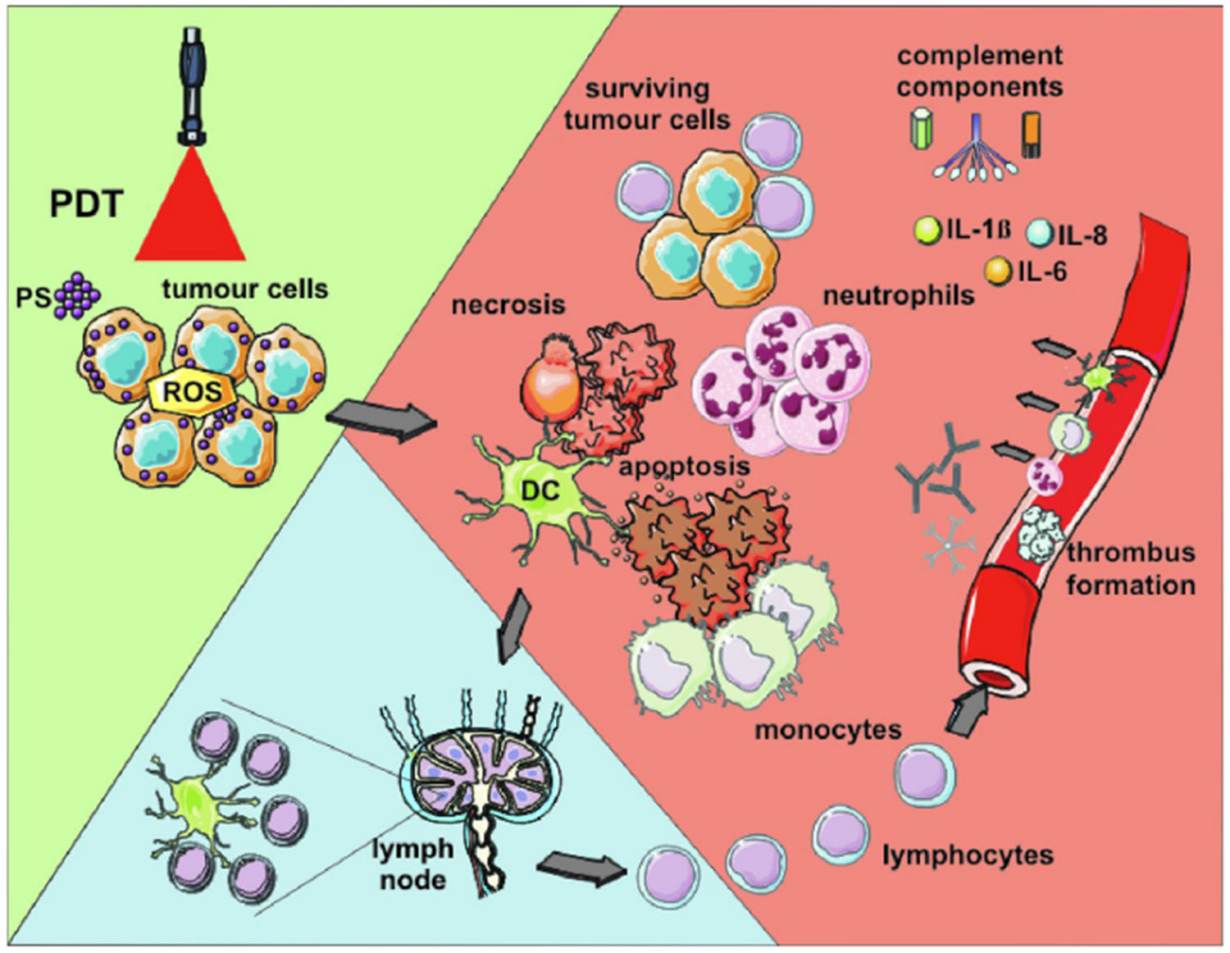
PDT-induced anti-tumor effects.^73^ Light-mediated excitation of the photosensitizer (PS) in the tumor leads to the production of ROS in the TME, resulting in tumor cell death (mainly *via* apoptosis and necrosis). Tumor destruction is further potentiated by damage to the microvasculature, which restricts oxygen and nutrient supply. Tumor cell death is followed by activation of the complement cascade, secretion of pro-inflammatory cytokines, and rapid recruitment of neutrophils, macrophages, and DCs. Dying tumor cells and tumor cell debris are phagocytosed by phagocytic cells, including DCs, which migrate to the local lymph nodes and differentiate into APCs. Tumor antigen presentation is followed by clonal expansion of tumor-sensitized lymphocytes that migrate to the tumor site and eliminate residual tumor cells.

Photodynamic therapy (PDT) is a well-established and minimally invasive cancer treatment modality involving the production of reactive oxygen species (ROS), such as singlet oxygen, responsible for cancer cell death by apoptosis. To be effective, PDT requires three elements: (1) Visible or near-infrared (NIR) laser light irradiation, between 400 and 700nm, (2) a molecule called photosensitizer (PS), and (3) an environment containing oxygen.^73^ PS usually are aromatic molecules able to absorb light and produce ROS when exited under irradiation at a specific wavelength in the presence of oxygen.^74^ Thus, PDT causes tissue damage, tumor cell toxicity, and immunogenic cell death (ICD), which subsequently allows for immune stimulation,^75^ as shown in Fig. 3. However, since the TME is a hypoxic environment, the therapeutic efficiency of PDT is limited.

To increase the efficiency of PDT in overcoming tumor hypoxic and immunosuppressive environment, PDT is often combined with other treatments. For instance, NPs have been used to modulate the TME by increasing the oxygen content in the hypoxic environment of the tumor. Liu *et al.* formed a nanocovalent organic polymer by crosslinking the PS meso-tetra(*p*-hydroxyphenyl) porphine (THPP), the anti-cancer drug cis-platinum (IV), and polyethylene glycol.^76^ When injected intravenously, NPs accumulate in the tumor, which increases oxygen content locally in the TME, hence increasing the effectiveness of PDT. Indeed, when comparing different treatment groups (control, NPs only, PDT only, and NPs + PDT), the animals treated with both light and NPs had significantly smaller tumors,^76^ indicating that nanomedicine and other oxygen-dependent treatments can be used synergistically to inhibit cancer progression.

#### 3.1.2. Photothermal therapy

PTT has drawn increasing attention as an effective and safe treatment method in oncology because of its minimally invasive and selective therapeutic potential.^77^ PTT is indeed a well-established cancer treatment modality involving NIR light and target-located light-absorbing agents to generate heat to kill cancer cells. Furthermore, the laser irradiation source can be external to the target tissues and applied topically or internal tissues can be irradiated by an interstitial fiber, depending on the depth of the target tissue. PTT-induced photothermal effects increase the tumor temperature and trigger immunogenic cell death (ICD).^78-80^ PTT can be applied by combining laser irradiation with locally administered NPs as light-absorbing agents. Nano-material-based PTT has especially shown promising results as a viable therapeutics for tumor ablation.^81-83^ Laser light can be precisely directed to the target tissues and light-absorbing agents allow for a localized increase in temperature in the target tumor, hence protecting surrounding healthy, NP-free tissues from a significant temperature change.^84^ Furthermore, laser irradiation with an adjustable dosage allows for the selective elimination of various types of cancers and minimizes damage to the surrounding nonmalignant tissues.^85,86^

PTT-induced ICD activates and directs the host immune system against tumors. Thus, immune cells can be activated to secrete cytokines that trigger systemic anti-tumor immune responses. PTT contributes to the treatment of metastasis^71^ and the formation of memory immune cells, hence potentially allowing for *in situ* cancer vaccinations.^87-89^

### 3.2. Nanoablative photoimmunotherapy and immune checkpoint inhibitors activate anti-tumor immunity and overcome the immunosuppressive TME

#### 3.2.1. Nanomaterial-based phototherapy

Many materials have been used in nanomedicine, such as liposomes, hydrogels, micelles, metallic NPs, and carbon nanostructures.^90,91^ Nanomedicine presents a strategy to combat the unique challenges presented by the TME due to the wide range of drugs and materials available as payload, enhanced timeliness, and efficiency of drug delivery, biocompatibility, and solubility compared to traditional medicine.^92-94^ NPs can carry a large variety of drugs, enzymes, and antibodies. Instead of being thwarted by the harsh conditions in the environment of a tumor, NPs strengthen immunological anti-tumor responses and modulate the TME to make other treatments more effective. Nanostructures offer new therapeutic alternatives for controlled drug delivery combined with imaging and treatment, such as targeted phototherapy. These combinatorial therapies can be applied to further modulate the TME, thus effectively controlling tumor growth, enhancing anti-tumor immunity, and extending overall survival.

**Fig. 4.**
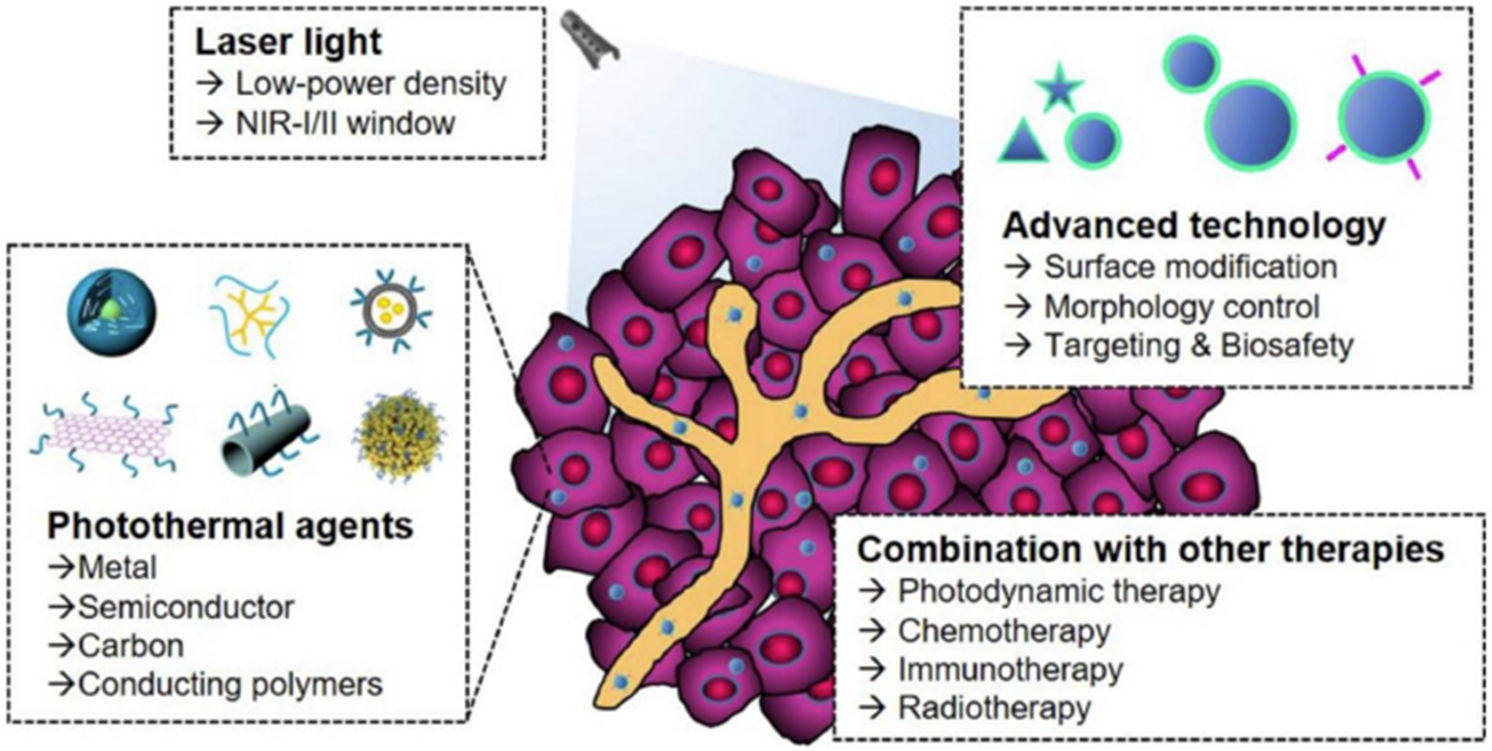
Components of the nanoablative photo-immunotherapies for cancer treatments.^77^

When used in combination with immunotherapy, the efficacy of nanomaterial-based PTT, or nanoablative photo-immunotherapy can be improved, potentially creating *in situ* anti-tumor vaccination.^95,96^ In general, ablation-based therapy can utilize different components to enhance the efficacy of the treatment, as shown in Fig. 4.

#### 3.2.2. Immunotherapy

The principle of immunotherapy relies on the stimulation of the immune system to prevent, control, and eliminate cancer.^97^ There are multiple forms of cancer immunotherapy, such as targeted antibodies,^98,99^ cancer vaccines,^100,101^ adoptive cell transfer,^102^ tumor-infecting viruses,^103^ immune checkpoint inhibitors,^104^ cytokine therapy,^105^ and adjuvants.^106^ Immunotherapy involves cooperation between immune cells in the TME. When tumor cells die, released tumor antigens and DAMPs stimulate robust immune responses against tumors, involving T cell priming and activation by mature DCs. Activated CD8^+^ T cells are tumor-specific and have anti-tumor functions. In short, one of the strategies of immunotherapy involves the presentation of tumor antigens by APCs, leading to the generation of specific anti-tumor CTLs.^107^

**Fig. 5.**
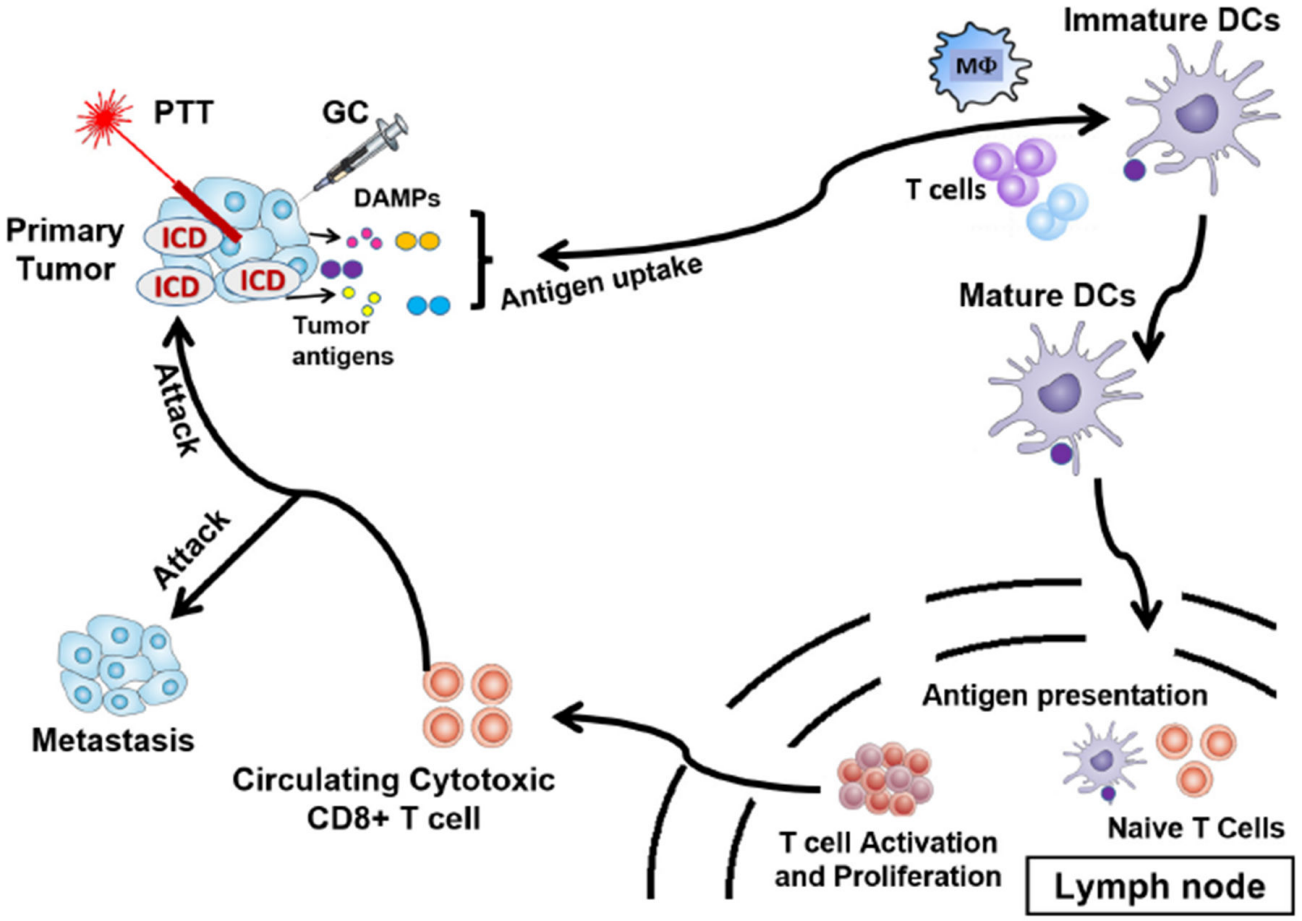
LIT induces a systemic, anti-tumor immunity for the treatment of metastatic cancers.^107^

Specifically, laser immunotherapy (LIT) is a combination of targeted PTT and immunotherapy.^77^ LIT combines a local intervention that specifically targets and kills tumor cells with laser irradiation and a local immunological stimulation to induce a systemic, anti-tumor immunity for the treatment of metastatic cancers, as shown in Fig. 5. *In vivo* results are very promising with significant tumor volume decrease and significant increase of animal survival. LIT is often combined with other cancer therapies. Using their drug delivery efficiency and NIR light-absorbing properties, NPs have been used to enhance the efficacy of LIT. Synergizing the effects of nanomedicine, ablation, and immunotherapy has been proven to be safe and effective for cancer treatment.^26^ The effects of LIT on different tumor types are actively studied in order to understand the mechanisms of immunotherapy and extend the use of LIT to as many cancer types as possible. The different elements of LIT are also under study with the objective of formulating optimal combinations.

#### 3.2.3. Immune checkpoint therapy

Although nanoablative photo-immunotherapy shows promising results, tumors have a multitude of immune escape strategies. For instance, if tumor cells stop expressing MHC-I on their surface, anti-tumor immune cells are no longer able to recognize them. Immunosuppressive molecules expressed on the surface of tumor cells can also prevent tumor killing. Furthermore, cytokines can influence the tumor-killing ability of T lymphocytes. Tumor cells can also decrease antigen presentation to T cells, decrease the number of T cell epitopes, induce Tregs activation, inhibit the proliferation and activation of CTLs, among others. It has been demonstrated that T cell checkpoint molecules, such as programmed cell death protein 1 (PD-1) and cytotoxic T lymphocyte-associated antigen 4 (CTLA-4) are frequently used by tumor cells to suppress anti-tumor responses of CTLs. Both tumor cells and immunosuppressing cells can express molecules that bind to PD-1 and CTLA-4 on the surface of T cells. PD-1 and CTLA-4 signaling in T cells inhibits effector function and cytokine production, effectively neutralizing their anti-tumor effects.

If the immune checkpoints PD-1 and CTLA-4 signaling in T cells are blocked, T cells can recover their effector functions. This is the reason why therapies such as nanomedicine, phototherapy, and immunotherapy can be combined with immune-checkpoint inhibitors (ICIs). Several studies showed that the combination of LIT with anti-CTLA-4 enhanced systemic anti-tumor responses and allowed for longer survival of 4T1 tumor-bearing mice. The most common ICIs used in the clinic to date are anti-PD-1 antibodies, such as pembrolizumab^108,109^ and nivolumab^110,111^ which bind to PD-1 receptors on T cells. Tremelimumab^112,113^ and ipilimumab^114,115^ are commonly used as anti-CTLA-4 antibodies. Anti-PD-L1 antibodies, such as avelumab^116,117^ and durvalumab^118,119^ can block PD-L1 interaction between DCs and tumor cells. As a result, T cell proliferation, production of cytokines and chemokines, as well as T cell cytotoxicity towards tumor cells, are augmented. This cancer treatment modality using checkpoint inhibitors is called immune checkpoint therapy (ICT).^120^ Disappointingly, ICT is usually not efficient enough, can be toxic,^121,122^ and patients can develop resistance to ICT.^123-125^

#### 3.2.4. Challenges of tumor immunogenicity

The effectiveness of immunotherapy and LIT depends on whether the tumor is “hot” or “cold”.^126^ ‘Hot’ tumors are immunogenic, i.e., they can be infiltrated with immune cells and have the potential to elicit an immune response to treatment. On the contrary, “cold” tumors are poorly immunogenic with very few infiltrating immune cells. Overall, the colder the tumor is, the poorer the prognosis.^127-129^

Therefore, generating anti-tumor immunity is not enough since the immunosuppressive effects of the TME still have to be overcome. The goal is to turn pro-tumor activities into anti-tumor pathways by recruiting immune cells and enhancing cancer cell immune recognition by directing the metabolic pathways towards a permissive TME. Another strategy that can be combined with promoting anti-tumor activities is to inhibit the pro-tumor metabolisms which prevent immune infiltration, survival, and anti-tumor functions. Combining PTT-induced ICD and ICT is actively investigated to target the immune subversive mechanisms of the TME.^130,131^

Even though PTT has been very effective in killing tumor cells, the effect of PTT in combination with other immunostimulatory drugs is currently under investigation, particularly on activating T cells and B cells. In the following sections, we review the central roles of T and B lymphocytes in response to phototherapy.

### 3.3. Phototherapy and T cells

T lymphocytes are adaptive immune cells designed to target and destroy pathogenic cells. There are several types of T cells, including CD4^+^, CD8^+^, and Treg cells. Tregs suppress other immune cells and can lead to tumor growth. The TME can actively repress T cell activation and functionality. One study utilized PTT to turn “cold” nonimmunogenic tumors into “hot” immunogenic TME, which increases T cell tumor infiltration.^132^ This study found that CD8^+^ and CD4^+^ T cell populations in PTT-treated tumors were 11.8 and 8.2 folds higher than the tumors in the control group. This indicates that PTT is an effective tool in activating T cells against cancer. Another study used PTT to target and destroy Tregs in the TME.^133^ Taken together, data showed that decreasing Tregs with this technique leads to smaller tumor volume and, depending on the treatment, enhances CD8^+^ T cell activation. These studies show that PTT has an immunogenic effect on the TME, leading to an increase in T cell infiltration.

Researchers have also developed chimeric antigen receptor (CAR)-T cells with recent approval from the US Food and Drug Administration (FDA) in 2017.^134^ This technique includes removal of T cells from the patient and genetic engineering of the T cells in the laboratory so that T cells can express chimeric antigen receptors for specific tumor-antigen recognition. This modification allows the genetically engineered T cells to recognize and selectively eliminate cells expressing the specific tumor-antigen after reinfusion of the T cells back into the patient.^135,136^ CAR-T cell therapy shows promising results; however, its efficacy in solid tumors remains an obstacle due to the physical barriers and the immunosuppressive TME.

**Fig. 6.**
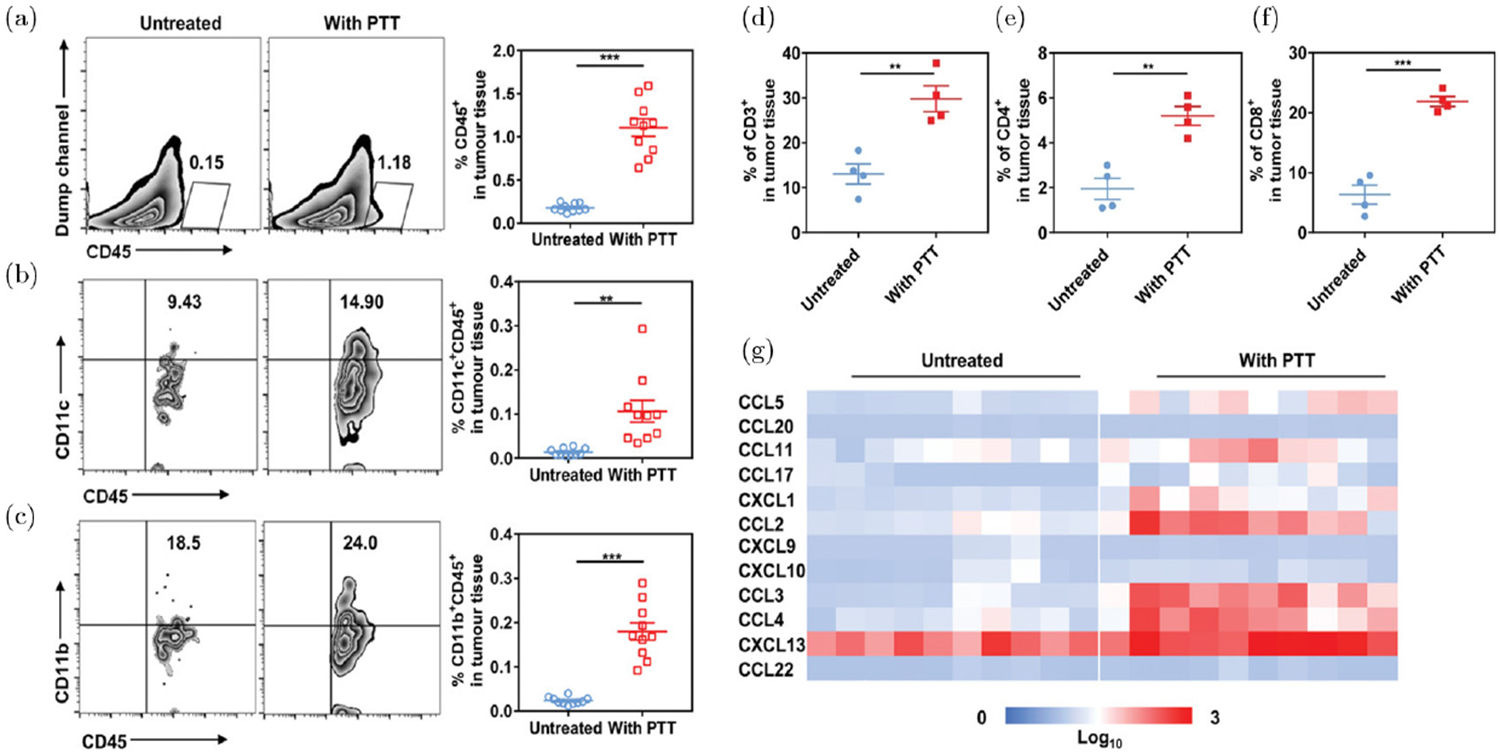
Effects of PTT on immune cell activities. ((a)–(c)) Expressions of infiltrating CD11c^+^ and CD11b^+^ immune cells in tumors after PTT. ((d)–(f)) Absolute frequency of the T cell markers CD3, CD4, and CD8 in tumor tissues after PTT administration. (g) Chemokine expressions in tumors. Adapted from Ref. 137.

To overcome the limitations inherent to immunotherapy caused by the immunosuppressive TME, synergistic treatments with PTT were investigated. For instance, Chen *et al.* utilized poly(lactic-co-glycolic) acid (PLGA) NPs loaded with the dye indocyanine green (ICG) in combination with PTT to increase tumor infiltration of the intravenously injected CAR-T cells and other immune cells^137^ (Fig. 6). After intratumoral administration of PLGA-ICG NPs in tumor-bearing mice, infiltration of monocytes and DCs was significantly increased after PTT as shown in Figs. 6(a)-6(c). Additionally, expressions of the T cell surface markers CD3, CD4, and CD8 within the TME were also increased after treatment (Figs. 6(d)-6(f)). Finally, expression levels of chemokines involved in immune cell recruitment such as CCL5, CCL2/3/4, CXCL1, and CXCL13 were also elevated in the TME after treatment with PTT + NPs (Fig. 6(g)).

In another study, Yu *et al.* developed a magnetic Fe_3_O_4_ nanoparticle (MNP) coated with myeloid-derived suppressor cell (MDSC) membranes (MNP@MDSC) to tackle immune escape by actively targeting the TME.^138^ When synergized with PTT, levels of HMGB1 and calreticulin (markers of ICD) and increased tumor-infiltrating CD8^+^ T cells were detected.^138^ Furthermore, Shen *et al.* designed TME-sensitive MnO_2_@ChitosanCyI (MCC) nanosystems in combination with PDT for 4T1 tumor-bearing mice treatment.^139^ Compared to MCC alone or laser alone, MCC + PDT significantly enhanced CTL infiltration into the tumor tissue. They found that MCC + PDT activated CD4^+^ helper T cells and CTLs and strongly inhibited the growth of both primary and metastatic tumors.^139^

Additionally, Lin *et al.* found that PTT, in combination with immunoadjuvant cytosine guanine dinucleotide (CpG) deoxynucleotides, showed increase populations of CD8^+^ T cells, IFN-*γ*^+^ CD8 T cells, and ratio of CD8^+^/CD4^+^ T cells in TILs.^140^ In another study, Zhou *et al.* administered N-dihy-drogalactochitosan (GC) immediately after PTT, resulting in upregulation of activated T cells in PTT + GC-treated tumor.^141^ For both of these studies, CD8^+^ T cell proportions were increased while Tregs proportions were decreased in untreated secondary tumors at distant sites. These studies suggest that the combination of PTT and immunotherapy can induce a systemic immunity against treated primary tumors as well as untreated distant tumors.

**Fig. 7.**
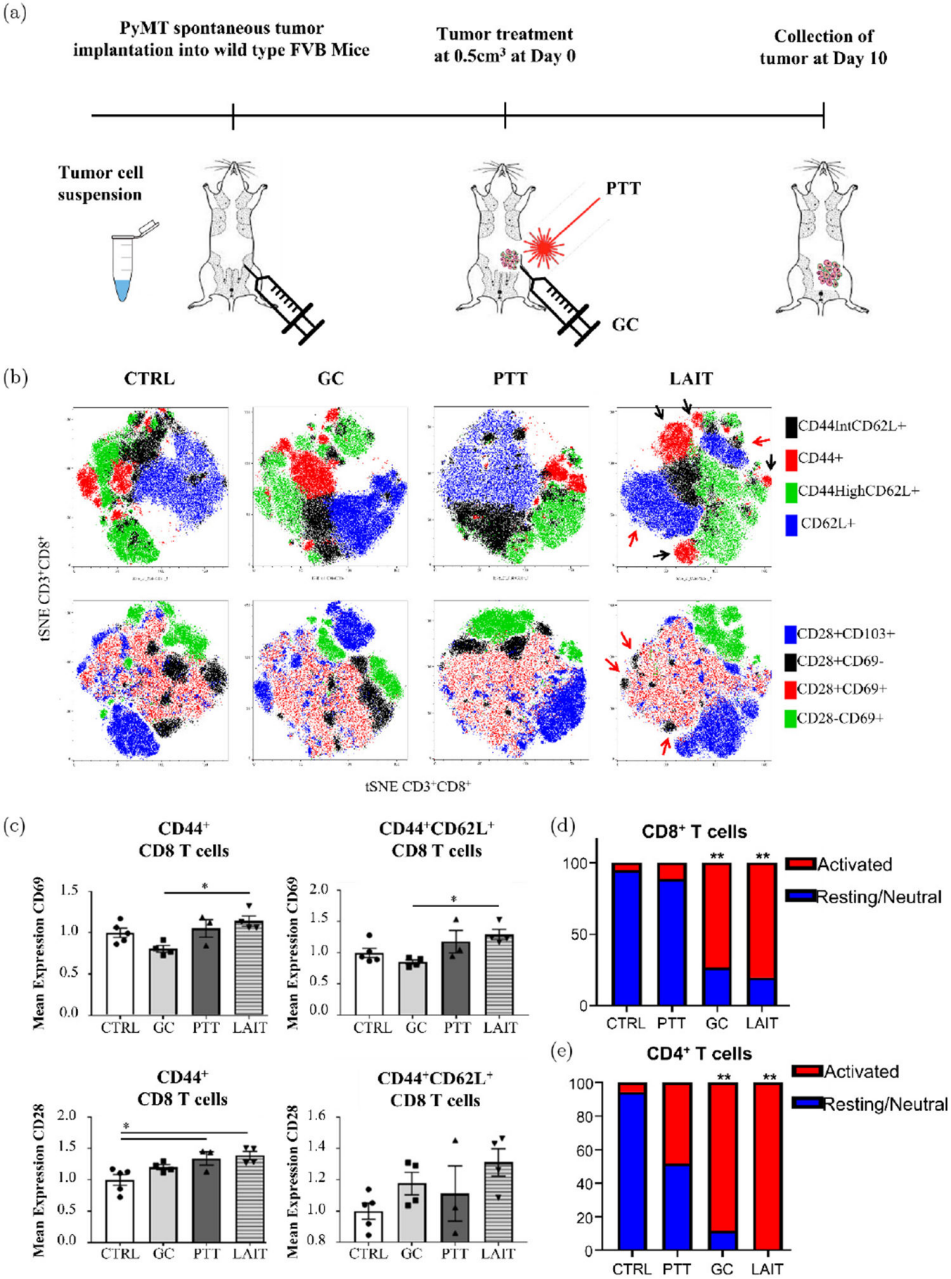
(a) Schematic of tumor implantation, treatment, and analysis. (b) T-SNE plots of tumor-infiltrating CD8^+^ T cells from different treatment groups. (c) Mean fluorescence intensity of CD69 and CD28 on CD8^+^ T cells. (d) Activated and resting CD8^+^ T cell proportions in different treatment groups: control, GC, PTT, and LAIT. (e) Activated and resting CD4^+^ T cell proportions in four different treatment groups. Adapted from Ref. 88.

Hoover *et al.* utilized localized ablative immunotherapy (LAIT) to investigate the contributions of CD4^+^ and CD8^+^ T cells to the therapeutic outcomes of LAIT using single-cell RNA sequencing (scRNA-seq).^88^ After implantation of murine mammary tumor virus-polyoma middle T (MMTV-PyMT) tumor cells (Fig. 7(a)), mice were divided into four different groups (untreated, PTT, GC, LAIT) when tumor volume reached 0.5 cm^3^. Analysis of the TME at day 10 after treatments revealed greater *T* cell diversity in LAIT-treated tumors as shown in the distribution and numerous distinct clusters in the *t*-SNE plot in Fig. 7(b). Expression levels of CD69 and CD28 in LAIT-treated tumors were both higher than the other three groups (Fig. 7(c)), and this signifies activation of CD8^+^ T cells as a response to LAIT. Additionally, a significant increase of activated CD8^+^ and CD4^+^ T cell proportions in GC and LAIT groups was observed (Figs. 7(d) and 7(e)). Overall, this study demonstrated that LAIT can enhance T cell activation and remodel the immunosuppressive TME.^88^

In summary, PTT can be used to assist T cell infiltration into tumors though a variety of different methods. While PTT itself can be used to invoke an immunogenic response, combinations of PTT with nanomedicine and immunotherapy have been shown to increase anti-tumor T cell responses.^142-145^

### 3.4. Phototherapy and B cells

T cells in the TME have been widely studied and therapeutically targeted in many cancer research studies. However, the role of B cells in cancer therapeutics remains unclear due to lack of studies pertaining to the effects of B lymphocytes on the TME. Tumor-infiltrating B cells are implicated in both pro- or anti-tumor immunities as observed by several authors.^146-149^ For example, Zhang *et al.* found that high intratumoral B cell infiltration was associated with increased Treg recruitment and expansion, leading to reduced infiltration of NK and CD8^+^ T cells in tumor sites.^150^ Another study performed by Li *et al.* demonstrated that adoptively transferred B cells promoted tumor-specific T cell immunity through IFN-*γ* and granulocyte-macrophage colony-stimulating factor (GM-CSF) production. This study showed that primed B cells can be capable of activating T cell immunity for tumor regression.^151^ These previous studies showed the dual functionality of B cell frequency and priming on tumor growth promotion and inhibition.

**Fig. 8.**
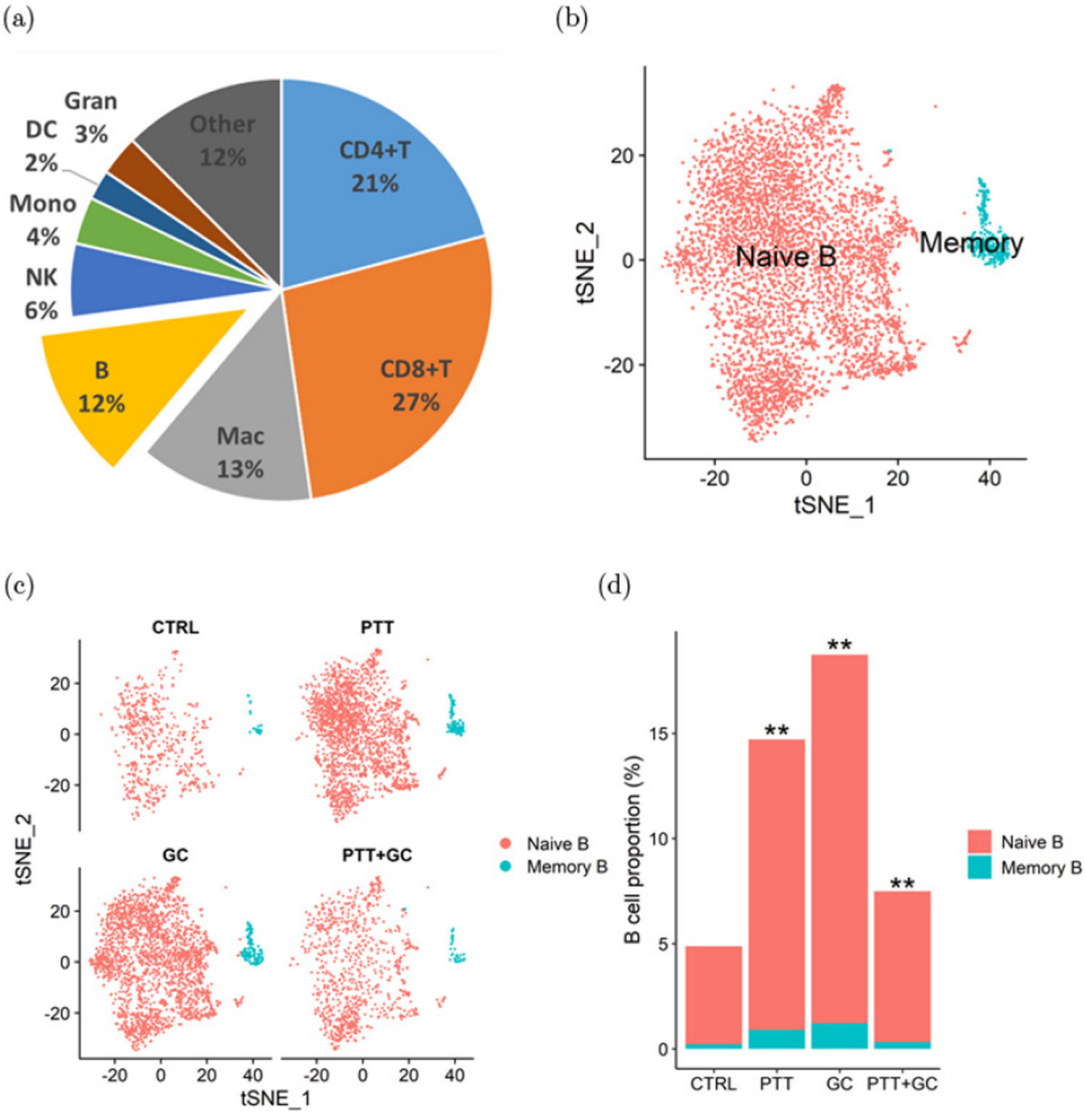
(a) Proportions of various immune cell types in MMTV-PyMT tumors. (b) B cell classification into naive and memory B cells using *t*-SNE plot. (c) *t*-SNE plots of B cells in control, GC, PTT, and PTT + GC treatment groups. (d) Naive and memory B cell proportions in four different treatment groups. Adapted from Ref. 89.

In order to investigate the subtypes and functions of B cells in the TME, Liu *et al.* utilized scRNA-seq to identify the different subtypes and activities of B cells in the TME after four different treatments: Control, GC, PTT, and PTT + GC.^89^ Figure 8(a) shows that 48% of TILs were CD4^+^ or CD8^+^, while only 12% account for B cell population. Out of the 49,380 CD45^+^ immune cells obtained, 5477 naive and 342 memory B cells were found and classified based on gene expressions encoding different B cell subtype markers (Fig. 8(b)). Naive and memory B cell populations were analyzed in the following treatment groups: Control, GC, PTT, and PTT + GC. Taken together, GC, PTT, and PTT + GC treatments increased the B cell proportions in tumor-infiltrating immune cells, suggesting that these treatments can influence the frequencies of TME-infiltrated B cells (Figs. 8(c) and 8(d)). Additionally, using analyses of gene functional enrichment and single-cell trajectory, Liu *et al.* concluded that GC and PTT + GC groups caused cell-state changes correlating with activation of B cells; however, only PTT + GC group induced a significant pro-inflammatory response. Overall, this study provided evidence that PTT+GC treatment resulted in greater activation of B cell gene signatures, higher expression of genes associated with antigen presentation, and higher degree of differentiation of naive B cells toward effector phenotype.^89^

In summary, while the functions of B cells in cancer treatment are still under debate, PTT in combination with immunostimulation, particularly as demonstrated in Ref. 89, could induce strong B cell-mediated anti-tumor immune responses. Therefore, B cells can play an important role in supporting anti-tumor immunity.^152^ However, more investigation on the functions of B cells in cancer treatment is needed, particularly on memory B cells for long-term anti-tumor immunity.

## 4. Clinical Relevance

### 4.1. T cells and TME modulation in response to phototherapy for improved clinical outcomes

Tumor-infiltrating T cells play a key role as anti-tumor effector immune cells. LAIT upregulated genes in CTL and helper T cell populations that positively correlate with prolonged survival of breast cancer patients by inducing broad pro-inflammatory responses and inhibiting suppressive signaling. In response to the combinatory treatment, augmented gene expression for anti-tumor interferon responses and T cell activation may provide beneficial effects for prognosis of breast cancer patients.

**Fig. 9.**
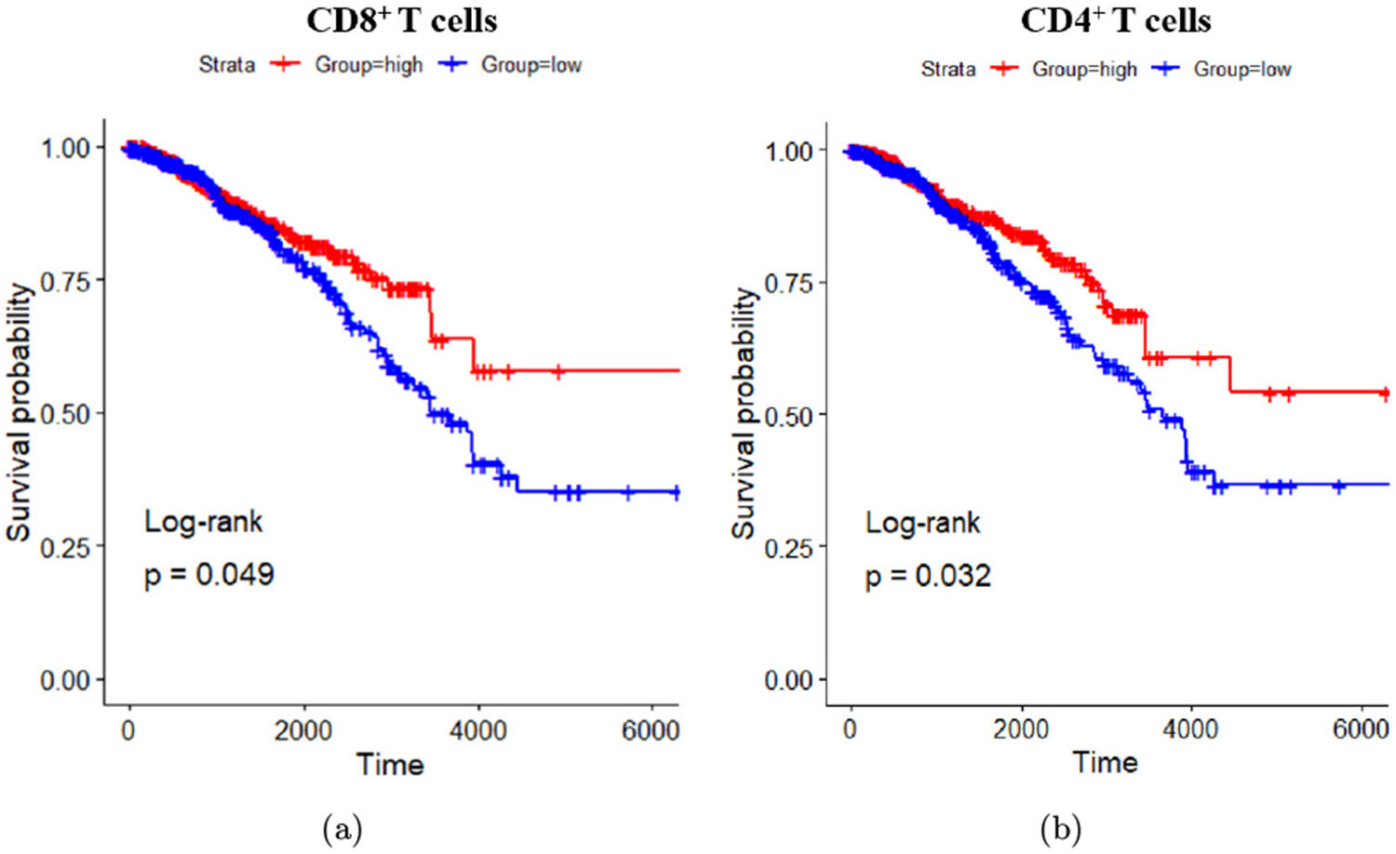
T cell-specific upregulated genes and breast cancer patient survival association. (a) Kaplan–Meier survival plots of patients with high and low expressions of LAIT-derived (versus GC-derived) upregulated genes in CD8^+^ T cells. (b) Kaplan–Meier survival plots of patients with high and low expressions of LAIT-derived (versus GC-derived) upregulated genes in CD4^+^ T cells. Survival time in days. Adapted from Ref. 88.

To investigate the clinical relevance of LAIT, Hoover *et al.*^88^ associated the expression of LAIT-derived (versus GC-derived) upregulated genes in mouse models with the survival in breast cancer patients. They were able to obtain an enrichment score (ES) based on gene set variation analysis (GSVA) after mapping the upregulated and downregulated genes in CD8^+^ and CD4^+^ T lymphocytes into their human counterparts. Using The Cancer Genome Atlas (TCGA) database, they found that breast cancer patients with higher ES scores based on GSVA of LAIT-derived upregulated genes in CD8^+^ and CD4^+^ T cells showed prolonged survival compared to patients with lower ES scores (Figs. 9(a) and 9(b)). Using the LAIT-derived downregulated genes, they found no effect on patient survival. These results suggest that LAIT-upregulated genes can provide favorable effects on prognosis. Overall, LAIT shows promising results in T cell activation and induction of advantageous upregulated gene sets for prolonged patient survival.^88^

### 4.2. B cells and TME modulation in response to phototherapy for improved clinical outcomes

Tumor-infiltrating B cells attract increasing interest for their anti-tumor activities even though B cells’ functions in cancer immunotherapy have yet to be understood. Recent studies showed that tumor-infiltrating B cells are positively correlated with favorable prognosis in many cancer types, especially breast cancer.^89^ PTT + GC-induced antibody production, antigen presentation, and secretion of pro-inflammatory cytokines make tumor-infiltrating B cells effector immune cells for tumor control and elimination. Elevated gene expression signatures associated with B cell activation, augmented antigen presentation, and increased interferon response genes are related to prolonged cancer patient survival and positive clinical outcomes.^89^

**Fig. 10.**
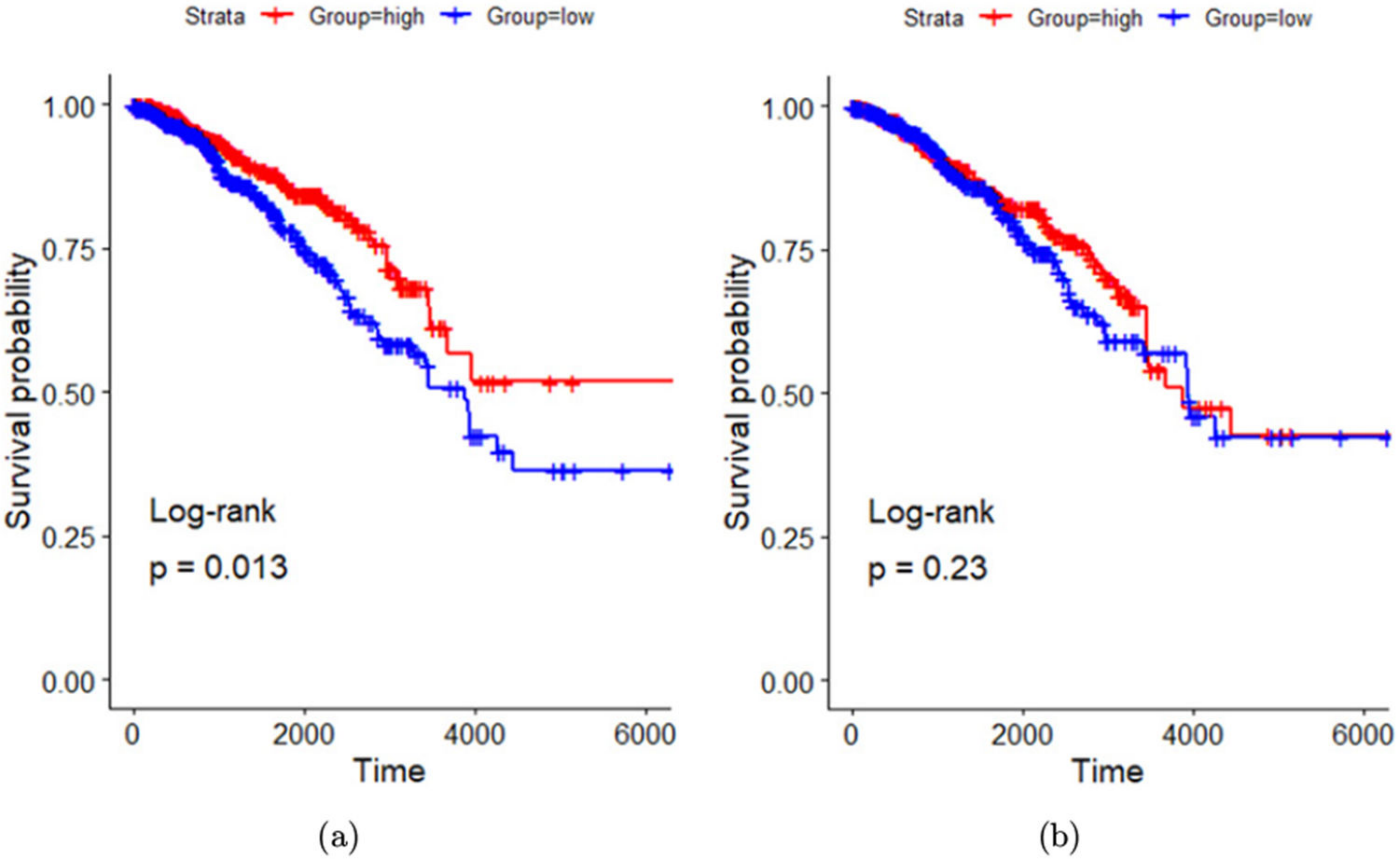
B cell-specific upregulated genes and breast cancer patient survival association. (a) Kaplan–Meier survival plots of patients with high and low expressions of PTT + GC-derived (versus GC-derived) upregulated genes in B cells. (b) Kaplan–Meier survival plots of patients with high and low expressions of PTT + GC-derived (versus GC-derived) downregulated genes in B cells. Survival time in days. Adapted from Ref. 89.

Similar to Hoover *et al.*, Liu *et al.* utilized the same method of obtaining enrichment scores after mapping both PTT + GC upregulated and downregulated gene sets in B cells. They found that breast cancer patients with higher expression of PTT + GC-derived upregulated genes exhibited prolonged survival compared to patients that showed lower gene expression as shown in Fig. 10(a). They also found no significant difference in survival time between patients with high and low expression of PTT + GC-derived downregulated gene (Fig. 10(b)). Overall, this study demonstrated that driving B cell activation using PTT + GC treatment has great potential in prolonging patient survival.

Those studies demonstrate the potential of activating T and B lymphocytes as well as remodeling the TME for clinical applications. Taken together, LAIT has great potential to prolong cancer patient survival by activating CD8^+^, CD4^+^ T cells as well as B cells. LAIT also elevated beneficial gene sets associated with greater survival of breast cancer patients. Overall, LAIT can rewire the immunosuppressive TME by establishing anti-tumor pro-inflammatory phenotypes in activated tumor-infiltrating T cells and B cells.

## 5. Conclusion

Progresses have been made in various cancer treatment modalities such as chemotherapy and immunotherapy against solid tumors. However, the immunosuppressive TME remains an obstacle to current cancer therapeutics. Phototherapy can induce ICD from tumor ablation and result in local and systemic anti-tumor immune responses. Its synergy with other cancer therapies results in the activation and differentiation of immune cells toward anti-cancer pathways. Therefore, multimodal treatments with phototherapy have the potential to overcome the pro-tumor TME. As discussed in this review, phototherapy can enhance tumor-infiltration of lymphocytes. Furthermore, using scRNA-seq, diversity and activation of T and B cells were measured in treated tumors. Phototherapy in combination with other treatment modalities provides a powerful platform to treat cancer through multiple approaches. LAIT, for example, has shown promising results in activating and enhancing anti-tumor immune responses through the differentiation of T and B lymphocytes into favorable subtypes. Overall, advancements in phototherapy have the potential to overcome the immunosuppressive TME through tumor cell killing, ICD induction, and lymphocyte infiltration and activation.

Further studies on the effects of phototherapy are needed, particularly on its immunological modulation, optimal applications, synergy with other therapies, in order to maximize its therapeutic effects and minimize the undesirable negative side effects, leading to an effective and safe modality for the treatment of poorly immunogenic metastatic cancers. Furthermore, the long-term effects of phototherapy, together with different immunotherapies, particularly the induced protective immune memories of both T and B cells, deserve in-depth studies in the future.
